# Supplementary material for: Fast and slow feedforward inhibitory circuits for cortical odor processing
Source: eLife. 2022 Mar 17;11:e73406. doi: 10.7554/eLife.73406 (PMC8929928; doi:10.7554/eLife.73406)
Supplement: Figure 7—source code 1. [file elife-73406-fig7-code1.zip › Figure 7 - source code 1/ReadMe.pdf]

Figure 7 – Source Code 1
"ReadMe"
Installation and Usage Instructions

Content of folders:

Figure 7 – source code 1
- ReadMe – this file

Figure 7 – source code 1 > Dynamic clamp source code
- Dynamic In Vivo.ipf – Igor procedure file (plain text)

Figure 7 – source code 1 > Sample data > Data for EPSG
- 29Apr4#1 006 EPSC.axgx – AxoGraph data file containing the EPSC episode and corresponding respiration episode measured from a SP cell *in vivo* in response to a 3 s-long application of ethyl-n-butyrate
- This file is read using *Dynamic In Vivo* and converted to a conductance template (EPSG) for use by the dynamic clamp

Figure 7 – source code 1 > Sample data > Data for interneuron APs > 1_NGla
- This folder contains AxoGraph data files for four NG cell-odor pairs, each containing the odor response episode and associated respiration episode
  - NGla 01Nov2 #1 008 – *response to lavender*
  - NGla 18Feb3 #1 010 – *amyl acetate*
  - NGla 18Feb3 #1 015 – *2-heptanone*
  - NGla 18Feb3 #1 018 – *1-heptanal*
- Each file is read using *Dynamic In Vivo*, then the spike timing is adjusted as summarized in the paper and detailed in the Igor code

Figure 7 – source code 1 > Sample data > Data for interneuron APs > 2_HZla
- This folder contains AxoGraph data files for three HZ cell-odor pairs in the same format as NG cells
  - HZ 13Mar3 #1 026 – *response to isoamyl acetate*
  - HZ 13Mar3 #1 028 – *1-heptanal*
  - HZ 21Feb3 #4 012 – *ethanol*
- Each file is read using *Dynamic In Vivo*, then the spike timing is adjusted as summarized in the paper and detailed in the Igor code

Detailed information about content and use of the Igor code:

(This text is also provided at the beginning of the *Dynamic In Vivo.ipf* file).

****
PURPOSE
****

This procedure runs a dynamic clamp to inject an EPSG template recorded 'in vivo' while simultaneously triggering an extracellular stimulator at times determined by APs recorded from NG and HZ cells 'in vivo'. The EPSC and AP data are read from AxoGraph files, assuming column 1 contains the EPSCs/APs and column 2 contains the respiration trace. The EPSC and AP episodes are aligned at the first upward transient in the respiration trace after odour onset, then the AP latencies in each respiration epoch of the AP episode are linearly morphed to match the corresponding respiration epoch of the EPSC episode. The EPSG and stimulator can be turned on or off as required. Note that the dynamic clamp runs only at 50 kHz, so the timebase of the sampled data (typically 20 kHz) has to be stretched to the higher rate.

****
SOFTWARE REQUIREMENTS
****

1. Igor Pro 6 or higher, available from https://www.wavemetrics.com/
2. ITC18 XOPs (Igor External Operations), available from https://www.heka.com/ and installed according to their instructions.

****
HARDWARE REQUIREMENTS
****

1. Instrutech ITC18 digitizing interface.

   NOTE: The successor to the ITC18 (HEKA LIH 8+8) has NOT been tested with this code.
2. MultiClamp 700A or B, or similar patch clamp amplifier.
3. Apple Mac computer with Intel chip or Motorola chip (PowerPC). This procedure has not been tested on a Mac with an M1 chip or on a Windows PC. Modifications to this code may be required to run on these computers.

****
DYNAMIC CLAMP
****

The dynamic clamp part of this procedure uses the command 'ITC18RunDynamicClamp' to calculate:

Io(t) = Ge*excitWave(t)*(E_Erev-Vm(t)) + Gi*inhibWave(t)*(I_Erev-Vm(t)) + Gc*forceWave(t)

where

Io(t) = command current to cell in Amps, from DAC0

Vm(t) = membrane potential of cell in Volts, into ADC0

excitWave(t) and inhibWave(t) contain the excitatory and inhibitory conductances (in Siemens), respectively

forceWave(t) is an additional forcing waveform

Gc, Ge and Gi are additional gains, set to 1 here

E_Erev and I_Erev are the excitatory and inhibitory reversal potentials (in Volts), respectively

The following waves are used by the dynamic clamp (where "EpiLen" is the length of the wave containing the data):

forceWave	Double precision, EpiLen length, contains forcing template
excitWave	Double precision, EpiLen length, contains EPSG template
inhibWave	Double precision, EpiLen length, contains IPSG template
DAC1Wave	Single precision, EpiLen length, DAC1 output, NOT USED HERE
DAC2Wave	Single precision, EpiLen length, DAC2 output
DIGWave	Single precision, EpiLen length, DIG0 output
sampWave	Single precision, EpiLen*4 length, entangled ADC0, 1, 2, 3

//• ADC3 and ADC4 are not used here. They can be used for sampling other signals.

Note that all of the above waves must be explicitly included in the ITC18 XOP command 'ITC18RunDynamicClamp' but only the excitatory conductance is applied here. Hence, the waves 'forceWave' and 'inhibWave' are set to zero (but must be defined and included in the argument list).

The following waves are used to obtain the results of the dynamic clamp:

VmWave	Single precision, EpiLen length, disentangled ADC0 from sampWave
IoWave	Single precision, EpiLen length, disentangled ADC1 from sampWave

Physical setup (NB: these channel assignments are hard-wired in the ITC18 XOP):

(i) DAC0 to COMMAND input on MultiClamp

(ii) ADC0 from SCALED OUTPUT on MultiClamp

(iii) ADC1 from RAW OUTPUT on MultiClamp (or can be used to sample other inputs, if desired)

(iv) DIG0 to stimulator

NB: Arguments to 'ITC18RunDynamicClamp' can be local variables, except E_ERev and I_ERev, which have to be globals.

****
USAGE
****

1. Install ITC18 XOPs (but code will automatically run in dummy mode if XOPs or hardware are absent).
2. Open this procedure in Igor and Compile.
3. Under Igor > Macros select 'Dynamic In Vivo'.
4. Use the GUI to control the procedure, as detailed below.

****
GUI
****

"Main" tab:

Evoke	Runs the dynamic clamp using the currently loaded excitatory postsynaptic conductance (EPSG) and stimulus waves. The start of each episode (default duration 10s) is indicated by 1 tone, the end by 2 tones. (In dummy mode the duration is very small). The dynamic clamp loops 'Num Epis' times. Press Command-period to stop prematurely. Note that, in a real experiment, the actions under the 'Setup' tab must be done first. See 'Setup' tab below for more details.
Episode	Episode counter (read-only).
RepInt (s)	Start-to-start time between episodes.
Num Epis	Total number of episodes to acquire.
Peak EPSG (nS)	Peak of excitatory postsynaptic conductance, calculated from EPSC data file (read-only). Value is set in "g's" tab.
Display Io	Checkbox to display the current injected by the dynamic clamp.
Save epis	Checkbox to save the Vm data. Data are saved (appended) at the end of each episode, so stopping prematurely does not lose any previous episodes.
# Saved	Number of episodes saved (read-only).
R	Reset file pointer to zero. This allows you to overwrite all previous data appended to the current file.
New File	Runs a dialog to open a new file.
Incr File	Automatically increments an integer at the end of the base file name. This assumes base file name has the format "Name.#" where # = an integer (typically start with '0').
Data File	Name of file in which the Vm data are being saved (read-only).
Test pulse	Opens a new window in which a test pulse is running. To exit this window, press 'escape' to stop the test pulse then click 'Exit'.
VC / IC	Select whether the test pulse is run in voltage clamp or current clamp mode.

"Gains" tab:

SampInt (µs)	Sample interval for dynamic clamp (DC), loaded EPSC episode, and loaded action potential (AP) episode with odour response of NG or HZ cell. These values are either obtained from the loaded data (EPSC, AP) or are hard-wired into the dynamic clamp command (DC).
EpiLen	Episode length (number of points) for DC, EPSC and AP data traces.
Vm Gain (MultiClamp)	'Scaled Output' gain for Vm set in the MultiClamp Commander.
ADC Range (ITC-18)	Programmable range of ADCs in ITC-18. For example, '10' means a range of +/- 10 Volts.
Stim Scale (nA/V)	'Stimulus Scaling' setting for IC mode in the MultiClamp Commander. The larger value (2 nA/V) is normally preferred for a dynamic clamp experiment in which large currents need to be injected.

"g's" tab:

EPSC Er (mV)	Assumed reversal potential for the loaded EPSC data. Used to calculate the conductance template (EPSG).
Conductance (nS)	Peak-scaled conductance of the EPSG template. This is normally adjusted at the beginning of the experiment to obtain the desired baseline firing response.

"Setup" tab:

Read EPSC File	Opens a dialog to read and plot the odour-evoked EPSC episode (and associated respiration episode) that is used to calculate the EPSG applied by the dynamic clamp.
EPSC resp threshold	Threshold value used by the procedure to find the peaks in the respiration episode that accompanies the EPSC episode.
Read AP File	Opens a dialog to read and plot the odour-evoked response of a selected NG or HZ cell, together with the corresponding respiration episode. These data are used for calculating the times at which to trigger the extracellular stimulator.
AP resp threshold	Threshold value to find the peaks in the respiration episode that accompanies each interneuron AP episode.
AP peak threshold	Threshold value (in mV) for detecting APs in the interneuron episode.
Morph	Runs a function that adjusts the timing of the APs in the interneuron episode in order to match the interneuron's respiration pattern to that of the EPSC episode. See paper and code for details.
Odour start (s)	Start time for odour onset in the 'in vivo' experiment.
Baseline duration (s)	Duration of baseline before odour onset, used to calculate the dynamic clamp conductance template.
DC episode duration (s)	Duration of entire dynamic clamp conductance template, and hence the duration of the recorded Vm episode.
EPSG ON	Checkbox to turn on the injected excitatory postsynaptic conductance (EPSG).
Stimulator ON	Checkbox to turn on the extracellular stimulator, which applies stimuli at times determined by the currently loaded interneuron data.
Plot DC Waves ON	Checkbox to display the EPSG and morphed stimulus trigger pattern that are applied.
Clear All Plots	Clears all plots opened in this tab, leaving just the main Vm plot.
